# The role of gene dosage in budding yeast centrosome scaling and spontaneous diploidization

**DOI:** 10.1371/journal.pgen.1008911

**Published:** 2020-12-17

**Authors:** Jingjing Chen, Zhiyong Xiong, Danny E. Miller, Zulin Yu, Scott McCroskey, William D. Bradford, Ann M. Cavanaugh, Sue L. Jaspersen

**Affiliations:** 1 Stowers Institute for Medical Research, Kansas City, Missouri, United States of America; 2 Department of Molecular and Integrative Physiology, University of Kansas Medical Center, Kansas City, Kansas, United States of America; The University of North Carolina at Chapel Hill, UNITED STATES

## Abstract

Ploidy is the number of whole sets of chromosomes in a species. Ploidy is typically a stable cellular feature that is critical for survival. Polyploidization is a route recognized to increase gene dosage, improve fitness under stressful conditions and promote evolutionary diversity. However, the mechanism of regulation and maintenance of ploidy is not well characterized. Here, we examine the spontaneous diploidization associated with mutations in components of the *Saccharomyces cerevisiae* centrosome, known as the spindle pole body (SPB). Although SPB mutants are associated with defects in spindle formation, we show that two copies of the mutant in a haploid yeast favors diploidization in some cases, leading us to speculate that the increased gene dosage in diploids ‘rescues’ SPB duplication defects, allowing cells to successfully propagate with a stable diploid karyotype. This copy number-based rescue is linked to SPB scaling: certain SPB subcomplexes do not scale or only minimally scale with ploidy. We hypothesize that lesions in structures with incompatible allometries such as the centrosome may drive changes such as whole genome duplication, which have shaped the evolutionary landscape of many eukaryotes.

## Author summary

Ploidy is the number of whole sets of chromosomes in a species. Most eukaryotes alternate between a diploid (two copy) and haploid (one copy) state during their life and sexual cycle. However, as part of normal human development, specific tissues increase their DNA content. This gain of entire sets of chromosomes is known as polyploidization, and it is observed in invertebrates, plants and fungi, as well. Polyploidy is thought to improve fitness under stressful conditions and promote evolutionary diversity, but how ploidy is determined is poorly understood. Here, we use budding yeast to investigate mechanisms underlying the ploidy of wild-type cells and specific mutants that affect the centrosome, a conserved structure involved in chromosome segregation during cell division. Our work suggests that different scaling relationships (allometry) between the genome and cellular structures underlies alterations in ploidy. Furthermore, mutations in cellular structures with incompatible allometric relationships with the genome may drive genomic changes such duplications, which are underly the evolution of many species including both yeasts and humans.

## Introduction

Multiple conserved processes act together to ensure eukaryotic cells maintain a stable chromosome composition, called the karyotype. Most organisms have a diploid karyotype with two copies of each chromosome. In nature, fungi are also commonly diploids, however, a haploid karyotype can be stably maintained in most lab strains [1, 2]. Changes in the karyotype through gains or losses of one or more chromosomes leads to aneuploidy, which is associated with miscarriage, cancer and fungal drug resistance [3–6]. Gains of whole sets of chromosomes (polyploidy) is another type of karyotype alteration that has driven evolution of many eukaryotes, including vertebrates and yeast such as *Saccharomyces cerevisiae* [7–11]. Increased ploidy is observed in certain highly differentiated human tissues such as liver parenchyma, heart muscle, placenta and bone marrow, and it is frequently observed in plants. However, polyploidy is also linked to aneuploidy as increased ploidy often leads to chromosome instability (CIN) [4, 12–15]. For example, in budding yeast the rate of chromosome loss in triploids and tetraploids is 30- and 1000-fold higher than haploids [16]. The mechanism(s) resulting in CIN in polyploids are poorly understood but may be linked to incompatible allometries (biological scaling relationships) driven by increasing genome size [16–19].

The cell division cycle is a highly conserved process that ensures chromosomes are replicated and segregated into daughter cells. Throughout eukaryotes, chromosomes are distributed into daughter cells by the mitotic spindle, a microtubule network formed around two spindle poles known as centrosomes in metazoans or spindle pole bodies (SPBs) in fungi. Duplication of the centrosome/SPB is coupled with the cell cycle such that cells entering mitosis have exactly two spindle poles to form a bipolar spindle [20, 21]. Errors in centrosome duplication result in the formation of monopolar or multipolar spindles. This has long been considered a driving factor in aneuploidy and polyploidy despite mechanisms to cluster multipolar spindles or surveillance mechanisms to detect spindle defects [22]. In *Saccharomyces cerevisiae* a mutant defective in SPB duplication was isolated by Lee Hartwell in his famous screen for cell division cycle mutants [23]. *cdc31-1* (allelic to *cdc31-2* used here) mutants arrest in metaphase due to monopolar spindles at the nonpermissive termperature of 37°C. Although the mutant was isolated in haploid yeast, viable *cdc31-1*/*cdc31-2* cells are diploid [24].

The formation of diploids in *cdc31-2* and other SPB mutants occurs at 23°C, a condition that is permissive for growth of the temperature sensitive mutant allele. Using classical genetic approaches, Schild, Ananthaswamy, and Mortimer 1981 showed that diploidization in *cdc31-2* was not linked to homothallism (mating-type switching) but to an early endomitotic event, likely a monopolar mitosis given the commonality of this phenotype in other SPB components and regulators [25–30]. Diploidization following chromosome segregation with a monopolar spindle is observed in mutants affecting the expression of SPB genes [27, 30], supporting the idea that SPB mutant proteins may lead to endomitosis and that amounts of various SPB components are highly regulated. While multiple SPB genes are haploinsufficient and/or toxic when overproduced, *CDC31* overexpression has only a mild effect on growth rate, and it is not haploinsufficient [31, 32].

Polyploidy may result from errors in chromosome segregation, but, somewhat paradoxically, increases in ploidy expand the burden of chromosomes that must be replicated and segregated by the cell cycle machinery. While polyploidy does not lead to the proteotoxic stress observed in many aneuploids [33, 34], genetic analysis of haploid, diploid and tetraploid yeast cells pointed to three processes that are essential for genome stability in cells of higher ploidy (tetraploids) but non-essential in cells of lower ploidy (haploids and diploids): homologous recombination, sister chromatid cohesion and mitotic spindle function [17, 35]. In yeast, where a single microtubule binds to each chromosome via its kinetochore [36], the number of microtubules must scale with ploidy. Consistent with this idea, the size of the SPB core, measured by electron microscopy (EM) as the diameter across its central region, increases linearly with ploidy [37–39]. How the SPB scales in size is unknown. The simplest idea, that polyploids have extra copies of SPB genes, seems insufficient as the SPB of haploid cells can also scale in size when the cell cycle is delayed or when the number of centromeres is increased [40, 41]. In addition, in SPB mutants that spontaneously diploidize, the cell must build a larger SPB and nucleate more microtubules–so it is unclear why the mutation would not result in another error in segregation that would further increase ploidy.

In metazoans, centrosome size also correlates with spindle size, and changes in its size have been linked to defects in chromosome segregation, aneuploidy and cancer [22, 42–44]. Here, we used budding yeast as a model system to examine the relationship between ploidy changes and SPB size scaling at a molecular level. We examined the diploidization associated with SPB mutants and performed a genetic screen to isolate suppressors of *cdc31-2* increase in ploidy. We found that spontaneous diploidization rescues the growth defect associated with some, but not all, SPB mutants. Mutations that are rescued by increased ploidy are only found in genes encoding specific SPB components that localize to regions of the SPB structure that we show do not scale linearly with chromosome number. We propose that diploidization acts as a ‘dosage’ suppressor and propose a model wherin acquisition of malfunctional centrosomes could drive eukaryotic evolution or disease progression by promoting changes such as whole genome duplication.

## Results

### Spontaneous diploidization in SPB mutants

**Fig 1:**
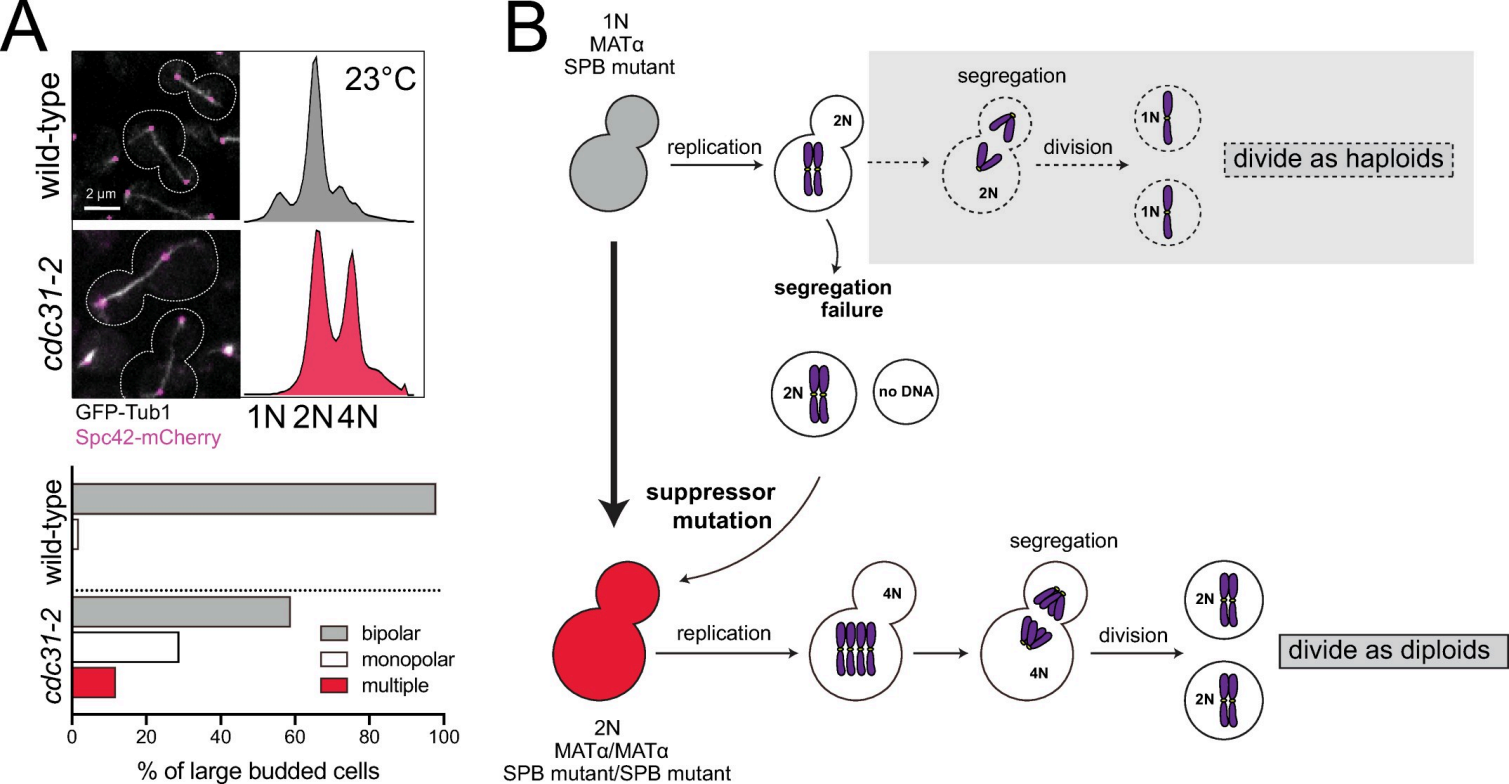
Spontaneous diploidization of SPB mutants such as *cdc31-2* at the permissive temperature. (A) Wild-type (SLJ7819) and *cdc31-2* mutant (SLJ10777) cells containing GFP-Tub1 (white) and Spc42-mCherry (magenta) were generated with a *pURA3-CDC31* plasmid. After growth on 5-FOA at 23°C to select for loss of the plasmid, the *cdc31-2* mutant spontaneously diploidize despite the formation of bipolar spindles. A representative image from each is shown along with the cell outline (dashes), and the percentage of large budded cells for with bipolar, monopolar or multipolar/broken spindles was quantitated (n>150). Bar, 2 μm. DNA content was assayed by flow cytometry. The biphasic peaks in wild-type cells represent cells with G1 (1N) and G2/M (2N) DNA content. At 23°C, *cdc31-2* mutants have diploid DNA content (2N and 4N). (B) Schematic of pathway to diploidization in *cdc31-2*. Cells containing *cdc31-2* do not undergo the typical cell division of haploids (gray box, dashed arrows). Instead, due to a defect in chromosome segregation, haploid (1N) cells undergo an aberrant cell division to produce a diploid (2N) and aploid (0N) cell. The diploid cell does not have the same defect as haploids, resulting in successful propagation. Because of this, we suspect that a suppressor mutation is acquired.

**Fig 2:**
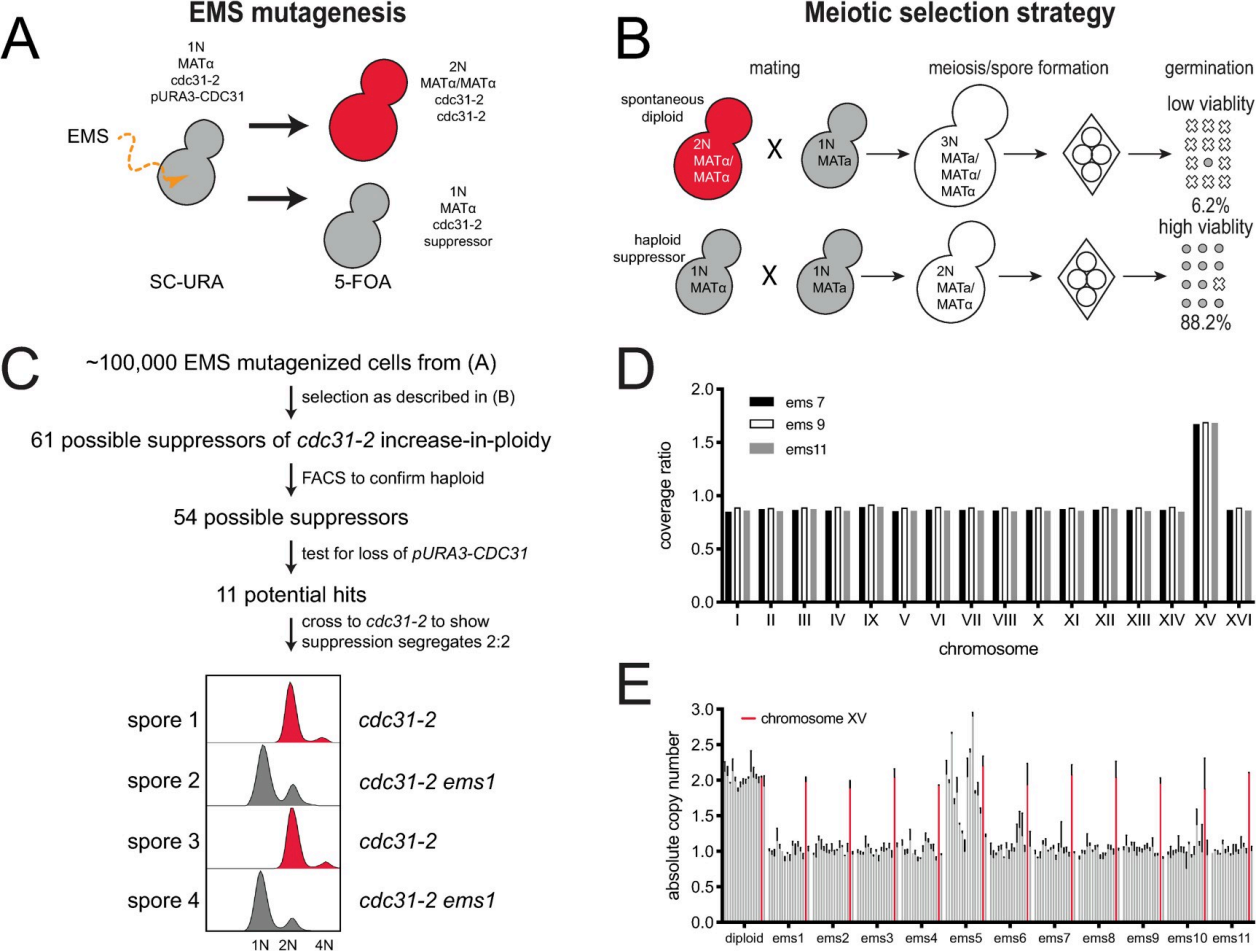
Screen for suppressors of *cdc31-2* diploidization. (A) Suppressors of the *cdc31-2* increase-in-ploidy were isolated following mutagenesis of SLJ6749 (*MATα cdc31-2 CAN1*::*KANMX trp1Δ*::*KANMX cyh2 LYP1 ura3-1 his3-11*,*15 ade2-1 pURA3-CDC31*) to ~50% viability using EMS. Loss of the *pURA3-CDC31* covering plasmid was selected using 5-FOA; strains without a suppressor will spontaneously diploidize as shown in Fig 1 while those with a suppressor will remain haploid. (B) Haploid (1N) or diploid (2N) strains can be mated to a haploid to form diploid (2N) or triploid (3N) cells. The viability of meiotic products is high from diploids (88.2%, n = 40 tetrads) compared to triploids (6.2%, 40 tetrads). Using this property, suppressors of diploidization were selected by mating to SLJ6750 (*MATa CDC31 can1Δ*::*STE2pr-HIS3MX CYH2 lyp1Δ*::*HYGMX ura3-1 trp1-1 his3-11*,*15 ade2-1*) on YPD + G418 + Hyg. Following sporulation, haploid selection was carried out using SD-His-Lys-Arg+canavanine+thialysine+cycloheximide. (C) From ~100,000 EMS mutagenized cells, 61 possible suppressors were identified, and 54 were confirmed to be haploids in a secondary screen of the original mutagenized colonies by flow cytometric analysis of DNA content. Of these, 43 appeared to have mutations in the covering plasmid that allowed for growth. The remaining 11 suppressors were analyzed by tetrad dissection to ensure that suppression segregates 2:2 through at least two crosses to SLJ6121 (*MATa cdc31-2 can1Δ*::*STE2pr-HIS3MX TRP1 CYH2 ura3-1 his3-11*,*15 ade2-1 pURA3-CDC31*). An example of flow cytometry data from one hit is shown. (D) Coverage ratio of all 16 yeast chromosomes in the haploid suppressors (*ems7*, *ems9*, or *ems11*) relative to the diploid control (*EMS7*, *EMS9*, *EMS11*). Other single nucleotide polymorphisms and insertions/deletion polymorphisms identified in the haploid suppressors are listed in S1 Table. (E) Quantitative PCR was performed on all 11 suppressors to determine the mean copy number of all 16 chromosomes relative to a wild-type, with chromosome XV plotted in red. Error bars, standard deviation from the mean.

Cdc31 is the yeast centrin ortholog, a small, highly conserved calcium binding protein present at centrosomes and other microtubule-organizing centers (MTOCs) across eukaryotes. A temperature-sensitive mutation in *cdc31-2* (E133K) causes haploid yeast cells to undergo spontaneous diploidization at the permissive growth temperature (23°C) immediately upon loss of a wild-type copy of *CDC31*, a phenotype we will refer to as increase-in-ploidy (IPL) [45]. The IPL phenotype is observed by flow cytometry as 2N and 4N peaks compared to the 1N and 2N peaks seen in haploid cells (Fig 1A). In agreement with classical genetic analysis [24], whole genome sequencing (WGS) of *cdc31-2* mutants shows that the IPL is an example of autopolyploidy, with two exact copies of each chromosome (diploid control in Fig 2D and S1 Table). No evidence of single nucleotide polymorphism was detected. Examination of spindle structure by fluorescence microscopy showed that 59% of *cdc31-2* large budded cells contained a bipolar spindle (Fig 1A and S1 Fig).

**Fig 3:**
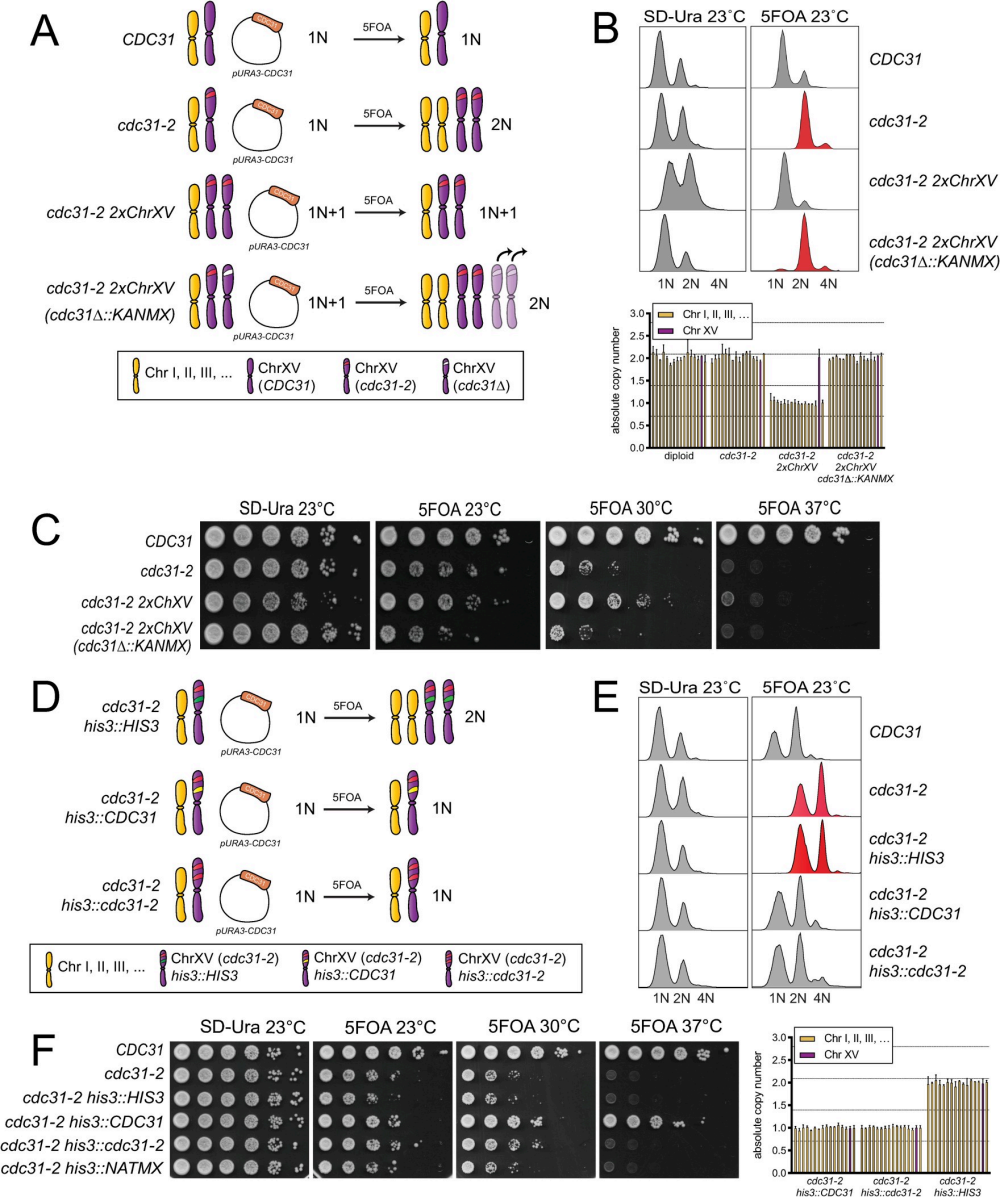
An extra copy of the *cdc31-2* gene is necessary and sufficient to suppress IPL. (A) To test if an extra copy of *cdc31-2* is necessary to suppress IPL, one copy of the *cdc31-2* locus was deleted in cells with a chromosome XV disome homozygous for *cdc31-2*, as illustrated in the schematic. *cdc31-2 ChXV(cdc31-2Δ*::*KANMX)* are predicted to form diploids with two extra copies of chromosome XV, however, qPCR and PCR analysis suggests that they revert to a diploid (2N) karyotype due to chromosome loss, as indicated. (B-C) The DNA content by flow cytometry (top) (B) and growth (C) of wild-type (SLJ7249), *cdc31-2* (SLJ809), the chromosome XV *cdc31-2* disome (SLJ7106, *cdc31-2 2xChXV(cdc31-2)*) and the deletion ((SLJ7111, *cdc31-2 ChXV(cdc31-2Δ*::*KANMX)*) that contain *pURA3-CDC31* were compared after growth in SD-Ura or 5-FOA at the indicated temperatures. Quantitative PCR was also used to verify the karyotype of strains from 5-FOA compared to a haploid control (bottom). Chromosome XV is plotted in purple. Error bars, standard deviation from the mean. (D) To test if an extra copy of *cdc31-2* is sufficient to suppress IPL, one additional copy of *cdc31-2* was inserted into the *HIS3* locus on chromosome XV. (E-F) The DNA content (E) and growth (F) of wild-type (SLJ7249), *cdc31-2* (SLJ809) and *cdc31-2* with an empty vector, wild-type *CDC31* or *cdc31-2* at *HIS3* (SLJ13092, SLJ13093 or SLJ13094) were analyzed after growth in SD-Ura or 5-FOA at the indicated temperatures. Quantitative PCR was used to verify the karyotype of strains from 5-FOA compared to a haploid control.

At 23°C, there is no evidence of *cdc31-2* progressing to tetraploids (Figs 1A and 3B) [24]. This raises an interesting paradox: if *cdc31-2* mutants diploidized via an endomitotic event due to a defect in cdc31-2 function (Fig 1B), why is that defect not perpetuated with the diploid cells undergoing an endomitotic event to form tetraploids? Instead, *cdc31-2* mutants continue to divide at 23°C as diploids over multiple generations, suggesting that the transition to the diploid state fully compensates for any microtubule nucleation defects. One possibility is the diploidization is genetically controlled through the acquisition of a suppressor mutation that bypasses the SPB defect caused by the original mutation, allowing *cdc31-2* mutants to divide as diploids (Fig 1B).

### Suppressors of *cdc31-2* spontaneous diploidization

To determine the mechanisms that drive the *cdc31-2* IPL phenotype and prevent further increases in ploidy, we developed a forward genetic screen to identify suppressors of the spontaneous diploidization observed in *cdc31-2* mutants (Fig 2A). Because *cdc31-2* is a recessive mutation, we maintained cells as haploids using a plasmid containing a wild-type copy of *CDC31* (*pURA3-CDC31*), known as a covering or complementing plasmid. Haploid *MATα cdc31-2 pURA3-CDC31* cells were mutagenized with ethyl methanesulfonate (EMS), individual mutagenized cells were selected, and the covering plasmid then was removed by growth on 5-fluoroorotic acid (5-FOA). Cells that contain an IPL suppressor are haploid while the remainder spontaneously diploidize due to the *cdc31-2* allele that is uncovered following plasmid loss (Fig 2A).

In budding yeast, the ability to sexually reproduce is not controlled by chromosome number but rather by the mating type locus (MAT) present on chromosome III. Typical diploids are heterozygous for MAT (*MATa/MATα*) and are therefore able to undergo meiosis to produce four viable haploid progeny known as spores. Triploid meiosis (*MATa/MATa/MATα* or *MATa/MATα/MATα*) is catastrophic because few spores contain chromosome combinations compatible with life. In our strain background, the viability of meiotic progeny from diploid meiosis in yeast homozygous for *cdc31-2* is 88.2% while the viability of progeny from triploids is 6.2% (Fig 2B). We therefore screened for suppressors of IPL through a selection scheme involving a non-mutagenized *MATa cdc31-2 pURA3-CDC31* strain mated with the EMS-induced mutant library. In this system, only *cdc31-2* cells that contain a suppressor of spontaneous diploidization will mate to form diploids, undergo a successful meiosis and generate viable progeny. In contrast, cells that spontaneously diploidized will mate to form triploids, which will die under the meiotic selection conditions (Fig 2B).

Of ~100,000 EMS mutagenized cells that were screened as described, we isolated 61 possible suppressors of the *cdc31-2* IPL phenotype (Fig 2C). To confirm that these cells had suppressed diploidization, we performed flow cytometry on all 61 hits. Of these, 54 displayed predominately 1N and 2N peaks characteristic of haploid cells and these were pursued further. Our system utilizes selection with 5-FOA, which is converted into a toxic metabolite in yeast containing *URA3* [46]. If a mutation is introduced into the *URA3* gene by EMS, haploid *cdc31-2* cells containing the covering plasmid would be able to grow on this counter-selection. To remove these potential false positives, we tested our 54 potential suppressors and found that 43 retained the covering plasmid. Further evaluation of these remaining 11 suppressor strains showed that each was linked to a single locus in the nuclear genome. Suppression of the *cdc31-2* IPL in one tetrad is shown in Fig 2C.

From these eleven suppressors, we chose three for further characterization by Illumina sequencing. Pooled genomic DNA from 20 meiotic progeny with and without the suppressor was analyzed. No single nucleotide or insertion/deletion polymorphisms were shared among all three strains, suggesting that suppression was not caused by a change in a single gene shared by all three mutants (S1 Table). A suppressor mutation within the genome also was not obvious. All contained variants in intergenic regions distal from promoter or terminator regions, and one contained a synonomous variant in *BUD27* (S1 Table). It is unlikely that any of these changes contributed to suppression of *cdc31-2* IPL. Strikingly, all three suppressors showed increased read depth for the entire length of chromosome XV relative to control when compared to all other chromosomes (Fig 2D). Thus, all three suppressors of *cdc31-2* IPL contained two complete copies of chromosome XV while retaining a single copy of all other chromosomes, possibly linking the dosage of a gene on chromosome XV to the bypass mechanism.

Using a quantitative PCR assay [47, 48], we determined that all eleven isolated suppressors have an increased copy number for chromosome XV (Fig 2E). Most contained a single copy of chromosomes I-XIV and chromosome XVI, however, one (*ems5*) had a more complex karyotype that may be due chromosome rearrangements such as diploidization followed by chromosome loss (Fig 2E). Alternatively, this mutant may exhibit cell to cell variation in chromosome content. Because all cells contained two copies of chromosome XV, this phenotype was further characterized as it suggests that disomy for chromosome XV can suppresses IPL of *cdc31-2*.

### Increased *cdc31-2* dosage suppresses diploidization

Cells disomic for chromosome XV exhibit a number of phenotypes, including a short delay in G1 phase of the cell cycle and a small increase in cell volume compared to normal haploid cells [49–51]. However, these phenotypes seem unlikely to be related to the mechanism of *cdc31-2* suppression since we did not recover disomies for other chromosomes with similar effects on cell size and the cell cycle. The specificity for chromosome XV suggests that suppression is linked to a gene or genes located on that chromosome, which are known to be upregulated in disomic strains relative to the rest of the haploid genome [52]. It seems likely that doubling the dosage of the gene(s) on chromosome XV is sufficient to alleviate the defect in SPB duplication that occurs in haploid *cdc31-2* mutants. The *CDC31* locus is located on the right arm of chromosome XV, making *cdc31-2* itself a leading candidate for dosage-mediated suppression.

To test the idea that *cdc31-2* itself suppresses IPL, we first tested if *cdc31-2* was necessary for suppression in the disomic strain (*cdc31-2 2xChXV*) (Fig 3A). Deletion of one copy of *cdc31-2* (*cdc31-2 2xChXV(cdc31Δ*::*KANMX*)) reduced growth at 23°C compared to the disomic control to a level similar of *cdc31-2* mutants (Fig 3A and 3C). These cells also spontaneously diploidized like *cdc31-2*. We anticipated that cells would contain four copies of chromosome XV, however, karyotype analysis using qPCR suggests that most viable cells have lost extra copies of chromosome XV, resulting in a diploid karyotype (Fig 3B). Nonetheless, these data are consistent with the hypothesis that the *cdc31-2* locus is necessary for suppression of IPL in the disomic strain.

Next, we integrated a single copy of *cdc31-2* containing its endogenous promoter, terminator and coding sequence into a covered haploid *cdc31-2* strain at the *HIS3* locus on chromosome XV to test if an extra single copy of *cdc31-2* is sufficient to suppress IPL (Fig 3D). We also constructed isogenic strains containing an empty vector or wild-type *CDC31* at *HIS3* as controls (Fig 3D). As shown in Fig 3E and 3F, a single extra copy of *cdc31-2* integrated into the genome in combination with *cdc31-2* at the genomic locus (*cdc31-2 his3*::*cdc31-2-HIS3*) is sufficient to suppress IPL observed in *cdc31-2* mutants after removal of the covering plasmid on 5-FOA. These cells grow as well, or better, than *cdc31-2* mutants that spontaneously diploidize or *cdc31-2* carrying a chromosome XV disome (Fig 3C and 3F). This could be due the fact that a single gene rather than multiple genes is altered, resulting in little or no change in the overall cellular proteome. Virtually identical results were also obtained if *cdc31-2* was added back at the *TRP1* locus on chromosome IV (S2 Fig). Taken together, these data demonstrate that an extra copy of the *cdc31-2* gene is both necessary and sufficient to suppress diploidization. The suppressor on chromosome XV is *cdc31-2* itself.

### SPB scaling along with ploidy change

**Fig 4:**
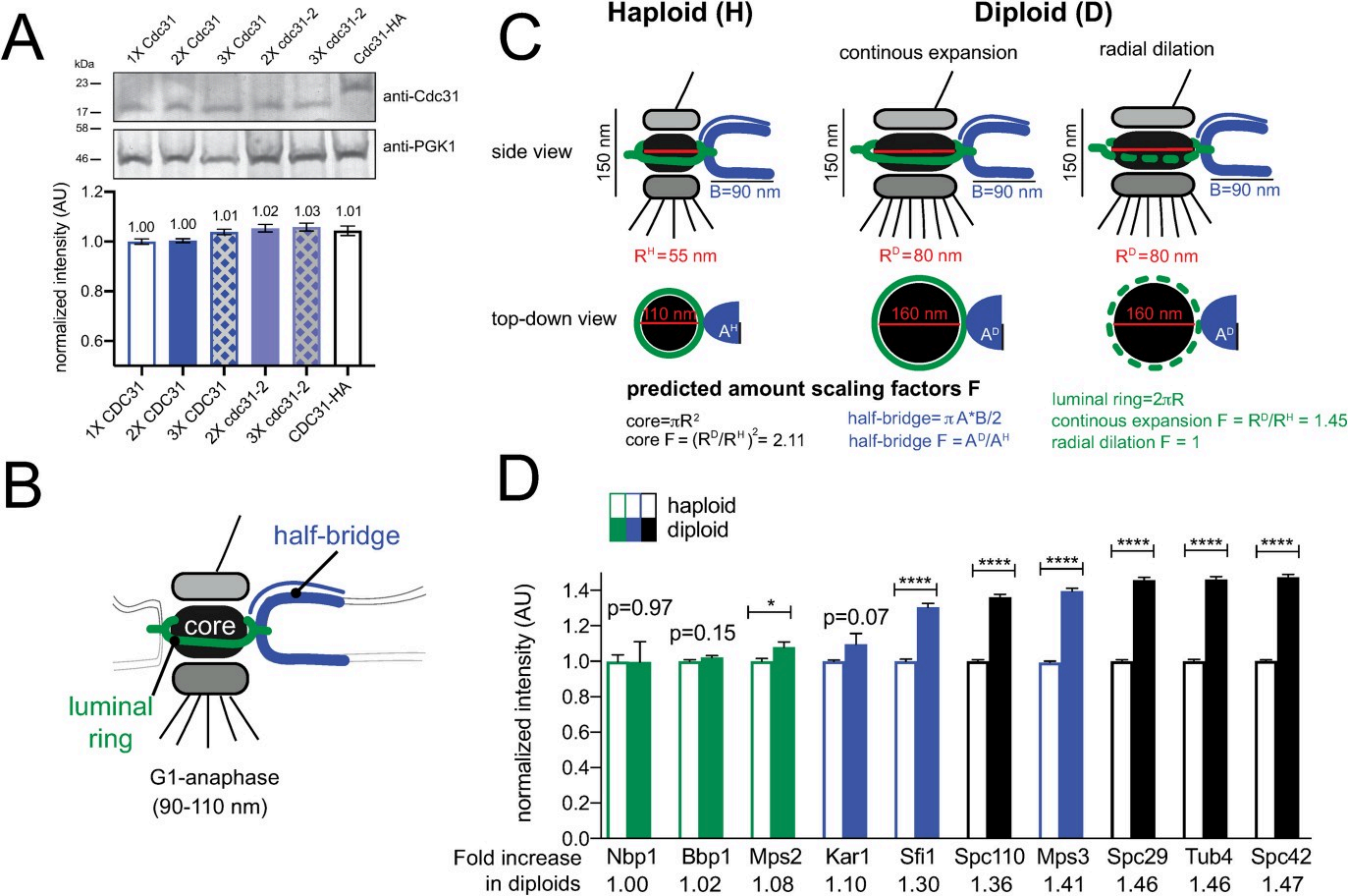
Scaling of SPB components with ploidy. (A) To determine levels of Cdc31 and cdc31-2, western blotting was performed using anti-Cdc31 antibody on strains containing the indicated number of gene copies of endogenously expressed *CDC31* or *cdc31-2*. Pgk1 served as a loading control. A representative western blot is shown along with quantitation from two replicates. Error, SEM. (B) Schematic of the SPB showing the location of the core, luminal ring and half-bridge. (C) Side and top-down views of the SPB from haploids and diploids along with dimensions reported from EM measurements. Assuming the SPB is round and the bridge elliptical and limited to a single protein layer, theoretical scaling factors can be calculated. Based on dimensions calculated from EM measurements, which are shown, a single layer of protein in the SPB core would be 2.11 times larger in diploids. The half-bridge is thought to be a monolayer of constant length in both haploids and diploids, however, its width may scale. Two potential models for scaling of the luminal ring are depicted: a continuous scaling, where components increase proportionally to the circumference of the SPB core (1.45-fold); or radial dilation, where the amount of components do not increase. (D) Levels of fluorophore tagged protein derivatives expressed from endogenous loci in haploids or homozygous diploids were determined by quantitative imaging (see S3 Fig). For each protein, levels in haploid cells were normalized to 1. Errors, SEM with N>300 for each sample.

Interestingly, while two copies of *cdc31-2* suppress IPL at 23°C, cells still arrest growth at 37°C (Fig 3C and 3F and S2 Fig). It is unlikely the mutant protein is unstable as Cdc31, like other small Ca^2+^ binding proteins, is extremely thermoresistant [53]. A western blot comparing levels of cdc31-2 to Cdc31 confirmed no significant change in protein levels (Fig 4A). While it is tempting to conclude that doubling the copy number of *cdc31-2* does not result in twice the protein, because Cdc31 localizes to multiple structures [54–56], it is still possible that two copies of *cdc31-2* may increase the amount of mutant protein available at the SPB. Unfortunately, we have been unable to localize protein using our antibody and we were unable to functionally tag Cdc31, similar to previous reports [27, 39, 57].

If the diploidization phenotype of *cdc31-2* were to be explained by ‘dosage suppression’, the structure formed by Cdc31 should be similar in size in haploids and diploids. In the electron microscope, the SPB appears as a trilaminar plaque-like structure (the core) embedded in the nuclear membrane by a pore-like structure (luminal ring) [37]. Associated with one side is a specialized region of the nuclear envelope known as the half-bridge, which is formed in part by Cdc31 (Fig 4B) [53, 58]. Although SPB diameter (measured at the SPB core) increases from 110 nm in haploid cells to 160 nm in diploids; the length and width of the half-bridge do not change (Fig 4B and 4C) [37, 39].

We tested the idea that SPB scaling underlies the diploidization of *cdc31-2* mutants by examining the correlation between protein levels at the SPB for different components and their associated mutant phenotypes (Table 1). Based on our model, we predict that levels of core proteins will be higher in diploids compared to haploids, as the size of the core, in theory, scales approximately two-fold between haploids and diploids. Because of this, we anticipate that viable core mutants must be haploid. While some mutants in core components may initially diploidize, because more protein is needed to build a larger SPB, IPL would not rescue and these alleles would be characterized as lethal alleles. Alternatively, ploidy should not affect levels of half-bridge proteins since its size is thought to be similar in haploids and diploids [39]. As a result, we anticipate diploidization would frequently be observed in mutants in these components as we observed for *cdc31-2* (Fig 4C).

**Table 1:** Ploidy level and function of *spb ts* alleles at 23°C in W303.

SPB *ts* alleles	Ploidy at 23°C	Localization at SPB*	References
*cdc31-2*	2N/4N	half-bridge	[24]
*CDC31-16*	1N/2N	half-bridge	[25]
*kar1Δ17*	2N/4N	half-bridge	[25]
*sfi1-3*	1N/2N	half-bridge	[93]
*sfi1-7*	1N/2N	half-bridge	[93]
*mps3-1*	2N/4N	half-bridge & luminal ring	[27]
*mps3-W477A*	2N/4N	half-bridge & luminal ring	[28]
*mps3-W487A*	1N/2N	half-bridge & luminal ring	[28]
*msp3-Y502H*	1N/2N	half-bridge & luminal ring	[28]
*mps3-A540D*	2N/4N	half-bridge & luminal ring	[28]
*mps3-F592S*	1N/2N	half-bridge & luminal ring	[28]
*mps3Δ2–150*	2N/4N	half-bridge & luminal ring	[63]
*mps2-1*	2N/4N	luminal ring	[94]
*mps2-381*	2N/4N	luminal ring	[28]
*ndc1-A290E*	1N/2N	luminal ring	[60]
*ndc1-39*	1N/2N	luminal ring	[60]
*ndc1-1*	1N/2N	luminal ring	[95]
*bbp1-1*	1N/2N	luminal ring	[96]
*nbp1-1*	1N/2N	luminal ring	[97]
*nbp1-ΔAH*	2N/4N	luminal ring	[29]
*cnm67Δ*	1N/2N	core	[98]
*spc42-11*	1N/2N	core	[99]
*spc29-3*	1N/2N	core	[100]
*cmd1-1*	1N/2N	core	[101]
*spc110-220*	1N/2N	core	[102]
*spc97-14*	1N/2N	core	[103]
*spc97-20*	1N/2N	core	[103]
*spc98-2*	1N/2N	core	[104]
*tub4-1*	1N/2N	core	[105]

* inner, central and outer plaque localization is denoted the core

Previously, quantitative fluorescence imaging has been used to determine levels of SPB components in haploid cells [59–62]. To test our hypothesis about SPB size and its link to diploidization, we compared the intensity of multiple SPB components endogenously-tagged with mTurquoise2 at the C-terminus in both haploid and homozygous diploid strains, with the exception of Kar1, which was tagged at the N-terminus (S3 Fig). SPB components clustered into three groups based on the intensity increase observed in diploids: no/mild (up to 1.1-fold), modest (1.1–1.4-fold) and major (over 1.4-fold) increase. Somewhat unexpectedly, none showed the anticipated increased fluorescence intensity based on scaling models and EM measurements (Fig 4D). For example, core SPB components (Spc110, Spc29, Tub4, Spc42), which underwent major scaling, showed a ~1.4–1.5-fold increase in diploids.

The group that showed a mild increase in diploids contained components of the luminal ring: Nbp1, Bbp1 and Mps2 (Fig 4D). The observation that this SPB substructure does not scale as predicted in our theoretical model could be caused by heterogeneity in ring shape [63] or by a difference in the actual mechanism of ring expansion. During post-mitotic nuclear pore complex (NPC) assembly, the membrane ring expands through a process known as radial dilation [64, 65]. A constant total amount of protein is spread over an expanding NPC core, decreasing protein area with the increase in size. Our data suggests that the luminal SPB ring expands by a similar radial dilation process (Fig 4D). The levels of half-bridge proteins Sfi1 and Kar1 showed a insignificant or modest increase in diploids (Fig 4D), similar to levels of Cdc31 that we observed by western blotting (Fig 4A). This suggests that the width of the bridge scales a small amount from haploids to diploids. Mps3 also showed an increase; as a dual component of the half-bridge and the luminal ring [63], this is consistent with bridge scaling (as seen for Sfi1 and Kar1) and radial dilation (as seen for Nbp1, Bbp1 and Mps2).

Overall, our measurements of SPB scaling are consistent with the idea that IPL is linked to SPB scaling: diploidization is frequently observed in multiple mutants in genes encoding components of the half-bridge and luminal ring that undergo limited or no SPB scaling, while diploidization is not observed in components of the SPB core, likely because dosage is insufficient given the increased amount of protein present in the diploid SPB (Table 1).

### Gene dosage as a general mechanism to suppress IPL in SPB mutants

**Fig 5:**
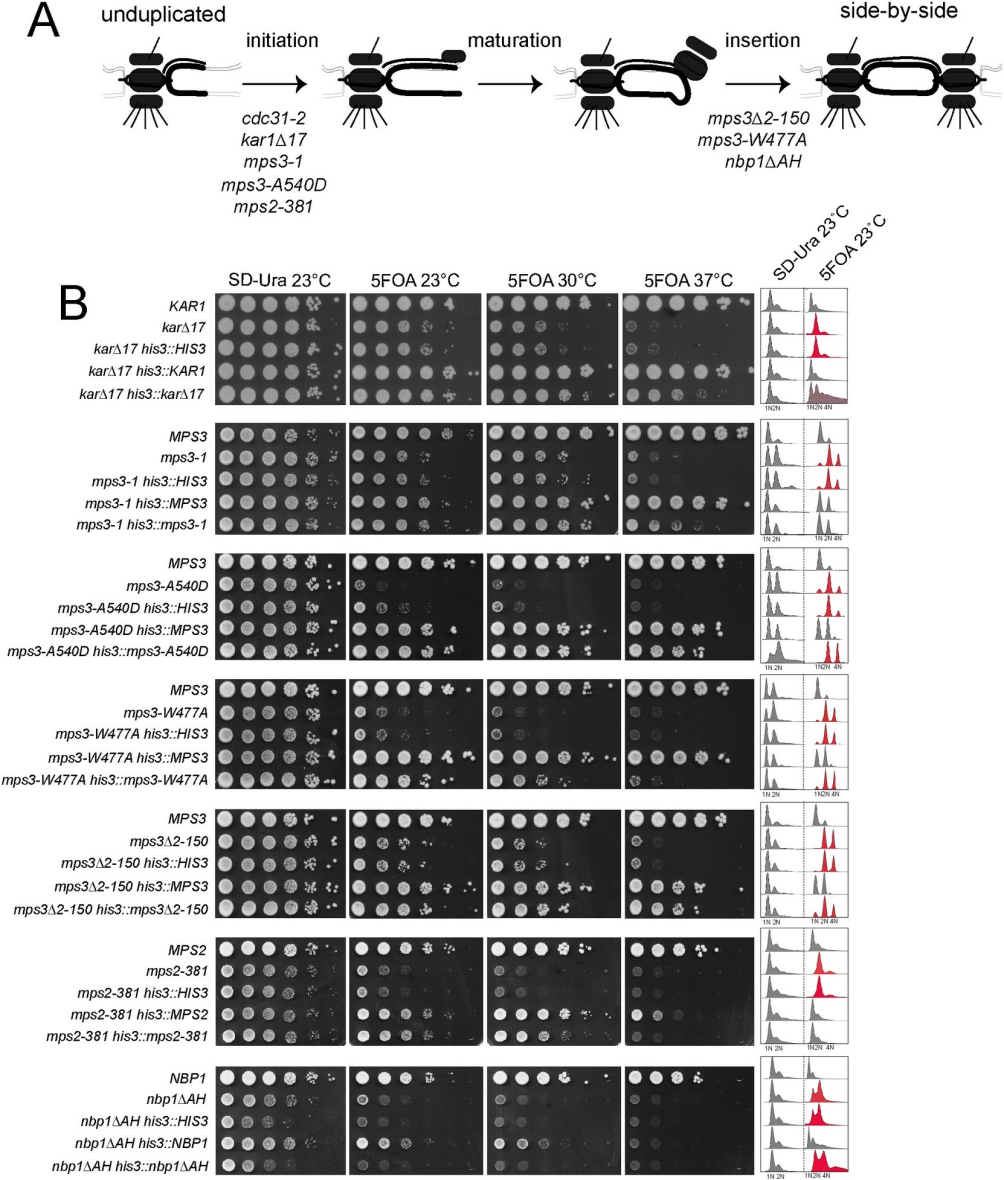
Dosage and IPL in other SPB mutants. (A) Schematic of SPB duplication pathway from an unduplicated SPB to duplicated side-by-side SPBs. Mutants defective in initiation, maturation and insertion of the new SPB have been isolated; shown are alleles required at each step that also exhibit IPL at 23°C (see Table 1). (B) To test if an extra copy of these mutant genes is sufficient to suppress IPL, one additional copy was inserted into the *HIS3* locus on chromosome XV as in Fig 3D, 3E and 3F. The growth and DNA content of wild-type, mutant and mutant with an empty vector, wild-type gene or mutant gene at *HIS3* were analyzed after growth in SD-Ura or 5-FOA at the indicated temperatures.

To determine if gene dosage is a general mechanism able to suppress SPB alleles, we were interested in determining if other mutants, like *cdc31-2*, would survive as haploids if an extra copy of the mutant gene was introduced at an ectopic site in the genome. We examined multiple mutants affecting different steps of SPB duplication (Fig 5A). *kar1Δ17* contains a partial deletion in the Cdc31 binding domain and arrests with a phenotype virtually identical to that of *cdc31-2* [25]. Using a covered haploid strain, we integrated a single extra copy of *kar1Δ17* into the genome of the *kar1Δ17* mutant at a marker locus. A single extra copy of *kar1Δ17* suppressed diploidization at 23°C in a small fraction of cells; however, most cells were of higher ploidy (Fig 5B). Next, we examined *mps3-1* or *mps2-381*, which have a SPB duplication defect similar to *cdc31-2* and, like *kar1Δ17*, show reduced Cdc31 recruitment to the half-bridge [27, 28]. An extra copy of *mps3-1* or *mps2-381* completely suppressed diploidization, similar to *cdc31-2* (Fig 5B). Interestingly, for *kar1Δ17*, *mps3-1* and *mps2-381*, growth fitness improved with doubled gene copy number at all temperatures, possibly suggesting that recruitment of half-bridge components is rate limiting in the mutants and the increased dosage of the hypomorphic protein rescues this process.

Examination of additional mutant alleles provided insights into the dosage-based diploidization survival mechanism. Three *MPS3* alleles (*mps3-1*, *mps3-A540D* and *mps3-W477A*) containing lesions in the C-terminal SUN domain that disrupt nucleoskeleton and cytoskeleton (LINC) complex formation exhibit different degrees of IPL suppression (Fig 5B). Increasing the dosage of *mps3-W477A* poorly suppressed its IPL phenotype despite the fact that this allele is expressed at levels higher than wild-type *MPS3* [28]. While the lack of IPL-based dosage suppression could be attributed to the allele, the fact that the *mps3Δ2–150* mutant also shows a similar phenotype points to another contributing factor. Unlike most *MPS3* alleles which are defective in initiation of SPB duplication, *mps3-W477A* and *mps3Δ2–150* block SPB insertion (Fig 5A) [63, 66]. Examination of two additional insertion mutants (*mps2-1* or *nbp1ΔAH*) showed that extra copies of these genes also did not rescue IPL or restore growth when added in extra copy (Fig 5A). Collectively, these data support the idea that the spontaneous diploidization serves as a dosage suppressor for specific SPB mutants that primarily affect initiation of SPB duplication.

## Discussion

Our goal was to understand the genetic control behind ploidy regulation of SPB mutants such as *cdc31-2* and to determine what, if any, benefits diploidization confers particularly in light of the fact that higher ploidy is associated with CIN. Our observation that an extra copy of *cdc31-2*, *mps3-1*, *mps2-381* and to a lesser degree *kar1Δ17* allows yeast containing these mutations to propagate as haploid cells strongly suggests that diploidization is a ‘dosage suppressor’. The fact that other SPB alleles (*mps3-A540D*, *mps3-W477A* and *nbp1-AH*) were not rescued by an additional copy of the mutant gene lends important insights into the suppression mechanism. First, the dosage-based spontaneous diploidization is limited to partial loss of function mutants that affect the first step of SPB duplication–half-brige elongation. Second, *cdc31-2*, *mps3-1*, *mps2-381* and *kar1Δ17* are defective in recruitment of Cdc31 to the half-bridge and share genetic interactions consistent with the idea that the hypomorphic allele is defective in half-bridge assembly [25, 27, 28, 53, 58]. As the half-bridge only moderately scales in size in diploid cells, increasing the copy number of the mutant gene coul eliminate defects in SPB function at the permissive temperature through additional recruitment of Cdc31 or other aspects of half-bridge assembly/stability.

Given SPB mutations are linked to chromosome segregation defects, an unresolved issue is why these alleles can be propagated as diploids under permissive conditions. Consistent with previous genetic analysis [24], our flow cytometry, WGS and karyotype analysis indicates that at a population level, *cdc31-2* is diploid. Formation of disomes or other aneuploids only appeared after treatment with a chemical mutagen and passage through meiosis. In contrast, mutations in α-tubulin or β-tubulin genes, which also affect chromosome segregation, result in disomy for chromosome XIII or II, respectively [67]. This suggests that diploidization of SPB mutants occurs through a non-stochastic event. Mutations in a number of pathways have been linked to spontaneous diploidization of haploid yeast strains, including: 1) mating-type mutations, which restores the diploid state through mating-type switching [68]; 2) cytokinetic mutants that fail in cell division to give rise to binucleate diploids [69]; 3) spindle mutants that result in errors in chromosome segregation to produce mononuclear diploids [45, 70]; and 4) histone mutants that disrupt centromere function [30, 71–73]. *cdc31-2* mutants do not undergo mating-type switching or display evidence of cytokinesis or centromere defects [74], so it is thought that diploidization arises due to an error in spindle formation, similar to chromatin mutants that have reduced levels of SPB components [30].

While lab strains of budding yeast are often maintained as stable haploid or diploid populations, polyploidy is common in natural yeast isolates [75]. The higher DNA state of triploid or diploid yeast allows for adaptation through the accumulation of mutations, some of which can be beneficial for fitness. A recent long-term evolution experiment using lab-derived strains indicated that diploidization is more prevalent than appreciated because lab growth conditions typically do not favor cells of higher ploidy [11]. Diploidization observed in *cdc31-2* and other SPB mutants is distinct from the diploid events seen in continuous culture: a hypomorphic allele likely results in a monopolar spindle, leading to an endomitotic event and diploidy, which is then a stable karyotype that can be propagated for generations [24]. In the original strain used by Hartwell and Mortimer (A364A/X2180-A), sporulation of a *CDC31/cdc31-2* heterozygous diploid resulted in four viable meiotic progeny. The *cdc31-2*-containing spores diploidized in less than 24 h following germination whereas *CDC31*-containing spores remained haploid [24]. This is nearly identical to the behavior we observed in W303, a strain containing a mutation in *SSD1*, an RNA-binding translational regulator, linked to aneuploidy tolerance in studies of natural yeast isolates [76]. Mutations in *KAR1*, *MPS3* and *MPS2* also show an IPL phenotype similar to *cdc31-2* in other lab strains with and without *SSD1* and other polymorphisms (W303, S288C, SK-1 and A364A), making it unlikely that sequence variants underlies spontaneous diploidization [23, 25, 27, 28, 53, 58, 77–81].

In previous work, incompatible allometry within the spindle was linked with the CIN phenotype in *S*. *cerevisae* and other fungi [82, 83]. Although the surface area of the SPB increases to expand microtubule nucleation capacity, the length of the pre-anaphase spindle does not change in tetraploids compared to diploids even though tetraploid cells have twice the DNA content. As a result, the incidence of syntelic (monopolar) chromosome attachments is higher in tetraploids [17]. The loss of chromosomes from tetraploid *C*. *albicans* is so dramatic that it results in diploid or near diploid progeny [84]. Unlike the SPB core, we show here that the luminal ring does not increase in size to a similar extent in diploids compared to haploids. This finding strongly suggests that, similar to the NPC, this region of the SPB expands and contracts via radial dilation, which alters protein density rather than protein abundance. This mechanism of scaling could facilitate cell cycle changes in the luminal ring size without the need to incorporate more protein. In haploids, the SPB core size increases from 90 nm in G1 to 110 nm in mitosis [37]; radial expansion of the luminal ring would accommodate this increase without addition of new protein. Similarly, as SPB size decreases back to 90 nm upon anaphase exit, radial contraction of the SPB would allow the luminal ring to shrink, without loss of protein components. The smaller G1 luminal ring would be denser than its mitotic counterpart, as both contain the same number of ring components distributed around smaller or larger diameter surfaces, respectively. Curiously, despite the constant size of the luminal ring, mutants that affect SPB insertion and the luminal ring do not show dosage-based scaling we describe here. We currently do not understand the molecular basis for diploidization of *mps3-W477A* and *nbp1-AH*.

In metazoans, centrosome function is regulated by factors involved in its duplication, maturation and microtubule nucleation. Centrosomal defects are linked to errors in chromosome segregation, in part due to the role of centrosomes in spindle organization. Our work further illustrates another possible role for centrosomes in driving genetic changes–the acquisition of mutations in genes such as centrin, which is conserved throughout eukaryotes, might promote stable genome amplification, including the genome duplications seen throughout evolution in fungi and metazoans or during tumorigenesis in humans.

## Materials & methods

### Yeast strains and plasmids

All strains are derivatives of W303 (*ADE2 trp1-1 leu2-3*,*112 ura3-1 his3-11*,*15 can1-100 RAD5+*) and are listed in S2 Table. Standard techniques were used for DNA and yeast manipulations, including C-terminal tagging with fluorescent proteins and gene deletion by PCR-based methods [85] (S3 Table). Single copy integrating plasmids containing SPB genes were made by PCR amplifying the open-reading frame, ~700 bp of promoter sequence and ~200 bp of the terminator from genomic DNA and assembling this DNA into pRG203MX [86] using Gateway assembly (S3 Table). Mutations were introduced by site-directed mutagenesis of the wild-type gene using the QuikChange mutagenesis kit (Agilent). Sequencing was performed to confirm correct base pair substitutions or deletions were made.

### Screen for *cdc31-2* IPL suppressors

SLJ6749 (*MATα cdc31-2 CAN1*::*KANMX trp1Δ*::*NATMX cyh2 LYP1 ura3-1 his3-11*,*15 ade2-1 pURA3-CDC31*) was grown overnight at 30°C in SC-Ura plus casamino acids to an OD_600_ of ~2.0. Cells were harvested and individual aliquots were mutagenized with a dosage of EMS that resulted in ~50% lethality compared to an untreated control. Following mutagenesis, cells were plated to YPD at 23°C at which time individual colonies were cherry-picked into 96-well plates to allow for automated pinning using the Singer ROTOR (Singer Instruments). Next, loss of the *pURA3-CDC31* covering plasmid was selected by growing cells on 5-FOA for 3 d at 23°C. Mating to SLJ6750 (*MATa cdc31-2 can1Δ*::*STE2pr-HIS3MX CYH2 lyp1Δ*::*HYGMX ura3-1 trp1-1 his3-11*,*15 ade2-1*) was performed overnight on YPD; mated cells were selected by growth on YPD containing 200 μg/ml G418 and 300 μg/ml hygromycin for 3 d. Cells were transferred onto sporulation media for 3 weeks at 23°C. Meiotic progeny were selected by two rounds of growth on SD-His-Lys-Arg containing 50 μg/ml canavanine, 50 μg/ml thialysine and 10 μg/ml cycloheximide for 3 d at 23°C. Suppressors of *cdc31-2* IPL give rise to colonies under these growth conditions.

From ~100,000 EMS mutagenized cells, 61 possible suppressors were identified. Flow cytometric analysis of DNA content showed that 54 exhibited peaks at 1N and 2N, which are typically observed in haploid yeast. Of these, we found using plasmid rescue, PCR and sequencing that 43 contained mutations in the *URA3* gene on the covering plasmid that allowed for growth on 5-FOA, thus these were false positives. The remaining 11 suppressors were analyzed by tetrad dissection to ensure that suppression segregates 2:2 through at least two crosses to SLJ6121 (*MATa cdc31-2 can1Δ*::*STE2pr-HIS3MX TRP1 CYH2 ura3-1 his3-11*,*15 ade2-1 pURA3-CDC31*).

### Whole genome sequencing (WGS)

Using 20 four-spored tetrads from a cross between an EMS-induced hit and SLJ6121, we identified the two progeny from each tetrad that were diploid (control) and the two progeny from each tetrad that were haploid (and therefore contained an ems hit). To ensure equal representation of colonies, each was individually grown, normalized by OD_600_, then mixed to achieve equal number of all 40 cells in the control and ems hit pools. Genomic libraries were made using the KAPA Library Preparation Kit (Roche) and prepared for paired-end sequencing on the Illumina MiSeq as previously described [87]. Reads were aligned to sacCer3 using bwa version 0.7.15-r1140 [88], and single nucleotide polymorphisms (SNPs) and insertion/deletion polymorphisms were identified using SAMtools version 1.5 [89]. SNP and insertion/deletion polymorphisms were annotated using snpEff version 4.3 followed by manual curation of SNPs present in W303 isolates [90]. Coverage was calculated using BEDTools version 2.25.0 [91]. In all cases, default parameters were used. Results are listed in S1 Table.

### Flow cytometry and qPCR karyotyping

DNA content was analyzed by flow cytometry in sonicated cells that had been fixed with 70% ethanol for 1 h at room temperature, treated with RNAse (Roche, Basel, Switzerland) and Proteinase K (Roche) for 2 h to overnight at 37°C and stained with propidium iodide (Sigma-Aldrich, St. Louis) in the dark at 4°C overnight. Samples were analyzed on a MACSQuant FACS Analyzer (Miltenyi Biotec) and data was displayed using FlowJo software (Tree Star, Ashland, OR). A wild-type haploid (1N/2N) and diploid (2N/4N) control were used to identify peaks, forward and side scatter were used to distinguish cell from debris, and pulse width (y-axis) by pulse area (x-axis) was used to distinguish single cells from doublets [92]. qPCR karyotyping was performed using centromere proximal primers for each chromosome arm as previously described [47, 48].

### Growth assay

To analyze growth phenotypes, 5 OD_600_ of cells from each strain were serially diluted 10-fold in SD media, and ~7 μl of each dilution was spotted on SD-Ura or SD plates containing 5-FOA (Sigma Aldrich). Plates were incubated at indicated temperatures for 2–4 days.

### Image analysis

Live cell imaging was used to study spindle structure in cells containing GFP-Tub1 (microtubules) and Spc42-Cherry (SPBs) using a Perkin Elmer (Waltham, MA, USA) Ultraview spinning disk confocal microscope equipped with a Hamamatsu (Hamamatsu, Japan) EMCCD (C9100-13) optimized for speed, sensitivity and resolution. The microscope base was a Carl Zeiss (Jena, Germany) Axio-observer equipped with an αPlan-Apochromat 100x 1.46NA oil immersion objective and a multiband dichroic reflecting 488 and 561 nm laser lines. GFP images were acquired with 488 nm excitation and 500–550 nm emission. mCherry images were acquired with 561 nm excitation and 580–650 nm emission. Data were acquired using the PerkinElmer Volocity software with a z spacing of 0.4 μm. Exposure time, laser power and camera gain were maintained at a constant level chosen to provide high signal-to-noise but avoid signal saturation for all samples. Images were processed using Image J (NIH, Bethesda, MD). A maximum projection image over relevant z-slices is shown in Fig 1A and S1 Fig. Cells were considered to be large-budded if the bud size was >30% the size of the mother cell.

Images for SPB intensity quantification in isogenic haploids and diploids were captured with a Nikon Spinning Disk controlled with NIS-Elements Viewer software equipped EMCCD camera and a PlanApo 100x 1.4 NA objective. Parameters, including laser power, exposure time, z-spacing and number of stacks, were set to identical value. Quantitation of levels of SPB proteins was performed with custom plugins (freely available at http://research.stowers.org/imagejplugins) written for ImageJ (NIH, Bethesda, MD). Prior to analysis, raw images were processed with background subtraction and summed to form a single plane image. Individual SPBs were identified using an ImageJ internal function “Find Maxima” and then chose “single points” as output. A circular ROI with a size of 7 pixel was drawn on each single point to cover individual SPB. Integrated intensity was then calculated on all ROIs. A sum projection image over relevant z-slices is shown in S3 Fig.

### Western blotting and quantification

Pelleted cells were washed in PBS and frozen in liquid nitrogen. Thawed pellets were resuspended in 1 ml lysis buffer (50 mM Tris, pH 7.5, 150 mM NaCl, 0.1%NP-40, 1 mM DTT, 10% glycerol and 1 mg/ml each pepstatin A, aprotinin and leupeptin) and ~100 μl of glass beads were added prior to bead beating for 1 min x 5 with 2 min on ice between beatings. Samples were spun at 5000 rpm for 2 min and the supernatant was transferred to a new tube. Protein concentration was determined using a NanoDrop Spectrophotometer (Thermo), and equivalent amounts of lysate were analyzed by SDS-PAGE followed by western blotting. The following primary antibody dilutions were used: 1:1000 anti-Cdc31 [27] and 1:5000 anti-Pgk1 (Invitrogen). Alkaline phosphatase-conjugated secondary antibodies were used at 1:10000 (Promega). Western blot band intensity was analyzed with ImageJ Gel quantification tool.

## Supporting information

S1 Fig Monopolar and multipolar spindles in *cdc31-2*. Example of a monopolar or multipolar spindle in the *cdc31-2* mutant (SLJ10777) cells containing GFP-Tub1 (white) and Spc42-mCherry (magenta). Bar, 2 μm.
(EPS)

S2 Fig An extra copy of the *cdc31-2* at *TRP1* is sufficient to suppress IPL. (A) An additional copy of *cdc31-2* was inserted into the *TRP1* locus on chromosome IV. (B-C) The DNA content (B) and growth (C) of wild-type (SLJ7249), *cdc31-2* (SLJ809) and *cdc31-2* with an empty vector, wild-type, *CDC31* or *cdc31-2* at *LEU2* (SLJ7104, SLJ7103 or SLJ7102) were analyzed after growth in SD-Ura or 5-FOA at the indicated temperatures.
(EPS)

S3 Fig SPB component levels in haploids and diploids. SPB components were fluorescently tagged with mTurquoise2 at their C-terminus, except for Kar1, which was N-terminally tagged. Each was expressed under the native promoter and imaged in haploids or homozygous diploids under quantitative imaging conditions (see Materials & Methods). Representative images of each haploid and diploid pair are shown with identical contrast adjustment. Note, the relative abundance between different SPB components cannot be inferred from this data as different settings were used for acquisition of samples. The diploid/haploid ratio is listed for each protein, based on quantitation in Fig 4D. Scale, 5 μm.
(EPS)

S1 Table Single nucleotide and insertion/deletion polymorphisms annotated with SnpEff for three *cdc31* suppressors. (XLSX)

S2 Table Yeast strain. (XLSX)

S3 Table Primers. (XLSX)
